# Return‐to‐play in athletes with transvenous and subcutaneous implantable cardiac defibrillator: A meta‐analysis

**DOI:** 10.1002/joa3.70131

**Published:** 2025-07-02

**Authors:** Rifqi Rizkani Eri, Sania Zahrani, Prasetyo Andriono, Haikal Balweel, Novaro Adeneur Tafriend, Agus Harsoyo

**Affiliations:** ^1^ Abdi Waluyo Hospital Jakarta Indonesia; ^2^ Gatot Soebroto Army Central Hospital Jakarta Indonesia; ^3^ Faculty of Medicine Universitas Indonesia Jakarta Indonesia

**Keywords:** arrhythmia, athlete, ICD, implantable cardioverter defibrillator, sudden cardiac death

## Abstract

**Background:**

Arrhythmia in athletes can be career‐threatening, and those with implantable cardioverter‐defibrillators (ICDs) face significant challenges in returning to play due to concerns about safety, efficacy, and arrhythmic risk. Since the last meta‐analysis, additional studies have been published, providing updated data that suggest both transvenous and subcutaneous ICDs (S‐ICDs) may allow for a safe return to sports through individualized decision‐making. This meta‐analysis aimed to reassess the safety and efficacy of ICDs in athletes returning to play.

**Methods:**

A systematic review and meta‐analysis were conducted following the PRISMA guidelines. Six cohort studies including 1183 athletes with ICDs were analyzed, with five of them on transvenous ICDs and one on S‐ICD. Primary outcomes included rates of appropriate and inappropriate shocks, shock‐related physical injury, cardiac adverse events, and sports discontinuation. Subgroup and sensitivity analyses were performed to explore heterogeneity.

**Results:**

The pooled rate of appropriate shocks was 13% (95% CI 0.11–0.16), while inappropriate shocks occurred in 4% (95% CI 0.02–0.11). No shock‐related physical injuries or cardiac adverse events during or shortly after sports were reported (0%). The rate of sports discontinuation was 2%, increasing to4% after sensitivity analysis. Transvenous ICDs showed lower inappropriate shock rates compared to S‐ICD.

**Conclusion:**

ICD use in athletes returning to play appears safe, with low adverse event rates and minimal sports discontinuation. These findings support tailored return‐to‐play decisions based on arrhythmia type, ICD programming, and psychological support, aligning with the 2024 HRS Class IIa recommendation.

## Graphical abstract

This meta‐analysis of six cohort studies (*n* = 1,183) found that athletes with transvenous or subcutaneous ICDs had 13% appropriate and 4% inappropriate shock rates, with no serious adverse events. Findings support individualized return‐to‐play decisions, considering arrhythmia type, ICD programming, and psychological support.

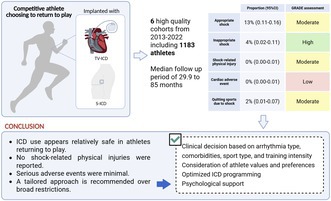

## 1 BACKGROUND

Arrhythmia poses a significant risk to athletes due to the threat of sudden cardiac death (SCD) during physical exertion.^1^ While they are often considered the healthiest group in the population, arrhythmia in athletes can be inherited, occur independently but worsen with physical activity, or result from vigorous training. Inherited arrhythmias such as hypertrophic obstructive cardiomyopathy (HCM), arrhythmogenic right ventricular cardiomyopathy (ARVC), or primary channelopathies like long QT syndrome (LQTS) and Brugada syndrome (BrS) put them at risk of tachyarrhythmic events, which may lead to the devastating sudden cardiac death. Several studies have also shown that athletes are more prone to acquired arrhythmias such as atrial fibrillation due to the heavy myocardial workload sustained over long periods, though this particular type of arrhythmia will not be the focus of this analysis.^1^, ^2^

When occurring during sport activities, cardiac events in athletes are devastating and may restrict them from returning to play for various reasons, primarily due to medical guidelines shaped by limited data on safety, as well as the psychological impact of the event. Over the last decade, implantable cardioverter‐defibrillators (ICDs) have offered a career‐saving treatment option, with the latest studies starting to showcase their safety and efficacy, particularly in preventing death during sports. Although most of the existing guidelines restrict athletes with ICDs from participating in competitive sports, recently, one particular consensus, the 2024 HRS Consensus on Arrhythmias in Athletes, grants athletes with ICDs a Class IIa recommendation in returning to play, which may allow them to return to competition with shared decision‐making.^3^, ^4^

This meta‐analysis aims to understand the safety and efficacy of ICDs in athletes choosing to return to play by compiling the latest available data and studies, including novel S‐ICD, which have contributed to the updated recommendations in the 2024 HRS consensus.

## 2 METHODS

### 2.1 Study selection

This meta‐analysis was conducted in adherence to the Preferred Reporting Items for Systematic Reviews and Meta‐Analyses (PRISMA) guidelines. Literature searching was performed in PubMed, PMC, SCOPUS, and Cochrane Library using the following search term: (athlete) and (implantable cardioverter‐defibrillator) from conception to April 15th 2024 with exact keywords displayed in Table S1. To ensure inclusion of the most recent evidence, an updated literature search was conducted during the study period, covering the interval from April 15, 2024 to May 12, 2025, using the same methodology.

Studies were eligible if they were Randomized Controlled Trials (RCT) or cohort (retrospective or prospective) in design and studied the mid‐term safety outcome of ICD with a minimum follow‐up duration of at least 1 year in active competitive athletes regardless of age. The studies must report at least one safety outcome, including device‐related outcomes: rate of inappropriate shocks and rate of appropriate shocks; clinical outcomes: shock‐related physical injury, rate of quitting sports due to shock, and cardiac adverse events. Cardiac adverse events are defined as incidents occurring during or within 2 h after sports participation, including: (1) tachyarrhythmic death or resuscitated tachyarrhythmia resulting from shock failure, incessant ventricular arrhythmia, or post‐shock pulseless electrical activity, and (2) severe injuries requiring hospitalization due to a shock‐related event or syncopal arrhythmia. Studies were excluded if they contained overlapping data, with only the most comprehensive dataset included, or if full‐text articles were irretrievable. No language restrictions were applied. The study selection process was conducted by two independent authors (R.R.E. and S.Z.).

### 2.2 Data extraction and study quality assessment

Full‐text articles were retrieved for studies that met the inclusion criteria. Data extraction and study quality assessment was independently performed by two authors (R.R.E. and S.Z.) utilizing a pre‐established standardized tabulated sheet. The authors extracted the following information: author, year of publication, region of study conduction, study design, population characteristic, definition of athlete, follow‐up duration, loss‐to‐follow‐up rate, baseline patient demographic and clinical characteristics, and previously described safety outcomes.

The study quality assessment was performed using the Newcastle‐Ottawa Scale (NOS) for non‐randomized studies. NOS is a star‐based scoring system with three main components (study group selection (4 points), comparability (2 points), and ascertainment of outcome of interest (3 points)) and a maximum score of 9 points for cohort studies. Studies with a score equal to or greater than 7 are generally considered to be of high quality. Each main outcome quality was assessed by the Grading Recommendations Assessment, Development and Evaluation (GRADE) tool, a tool for evidence quality assessment and strength‐of‐evidence recommendation. All discrepancies in study selection, data extraction, and study quality assessment were resolved through discussion with co‐authors (P.A., H.B., N.A.T., A.H.) consulted to reach a consensus.

### 2.3 Data synthesis and statistical analysis

The meta‐analysis was conducted using RStudio (version 2024.09.1) with the ‘meta’ and ‘metafor’ packages. Pooled effect sizes and corresponding 95% confidence intervals (CIs) were calculated using a random‐effects inverse variance model, selected to account for anticipated heterogeneity across the studies. Heterogeneity was assessed using the *I* ^2^ statistic, with *I* ^2^ ≥ 50% indicative of substantial heterogeneity. In the presence of significant heterogeneity, potential sources were explored via subgroup analyses and meta‐regression. Given the limited number of included studies, if meta‐regression or subgroup analyses were deemed inapplicable, sensitivity analysis utilizing the leave‐one‐out method was performed to examine the influence of individual studies on the overall pooled effect size and heterogeneity. Forest plots were generated to visually represent the safety outcomes across the included studies.

## 3 RESULTS

### 3.1 Search results

**FIGURE 1:**
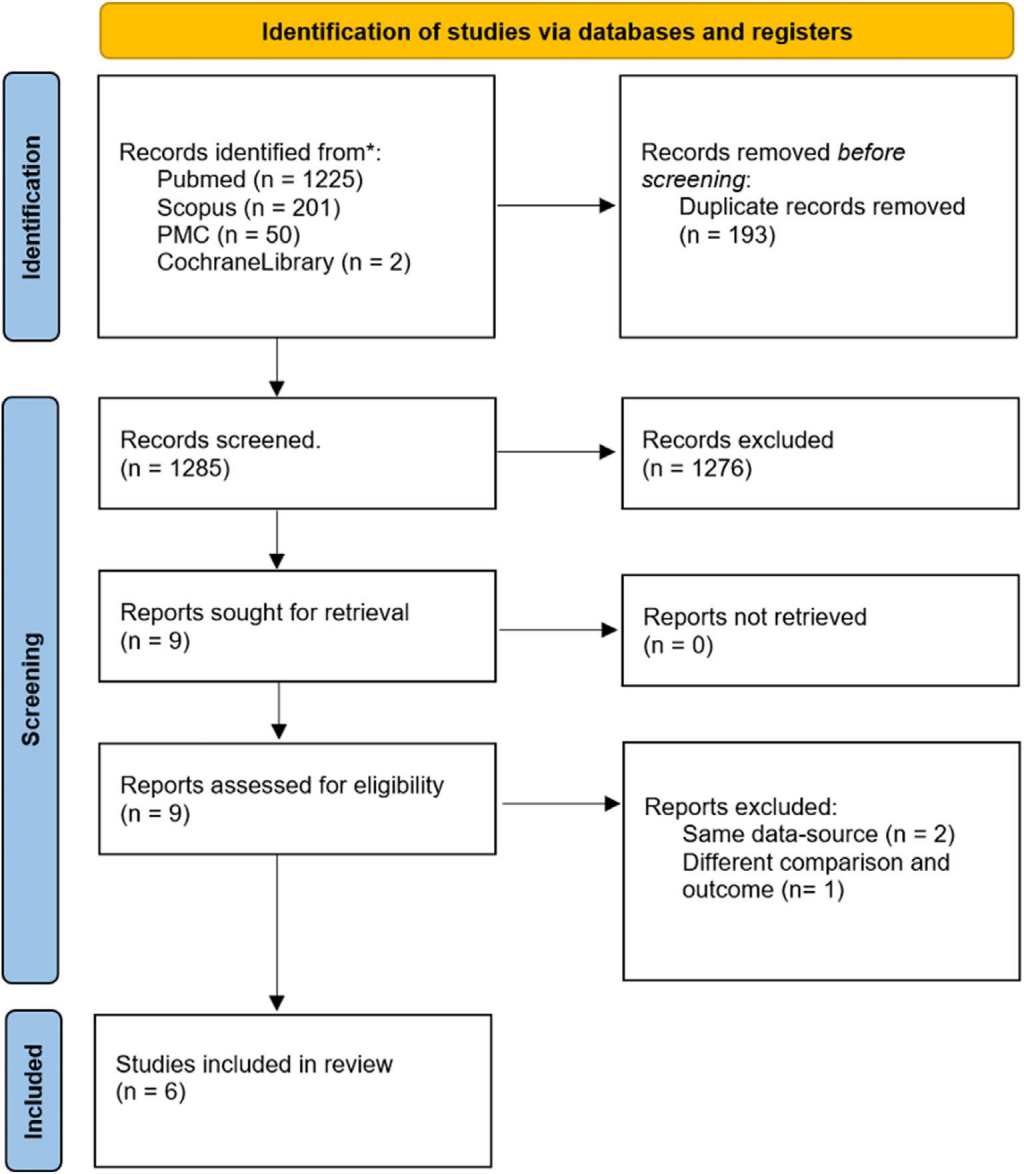
PRISMA flowchart of searching strategy.

A total of 1478 articles were identified from the database searching, including 1461 articles from the initial searching and 17 articles from the updated searching. After the removal of 193 duplicates, 1285 articles were left for title and abstract screening. After title and abstract screening, 9 studies were included for the full‐text review. Three studies used data from the same database, leaving the study with the most complete and updated data included for the analysis (Lampert et al., 2017).^5^ One study was excluded due to a different patient comparison and outcome of interest, leaving six studies for the final meta‐analysis. One of these six included studies (Gasperetti et al., 2024)^6^ was added from additional literature searching.^6^ The full study selection process is illustrated in Figure 1.

### 3.2 Risk of bias

The six included studies were of low risk of bias, with NOS scores ranging from 7 to 8 (Table S2). All studies controlled for key variables, but possibly confounding additional factors were not consistently accounted for due to the nature of the cohort. Two studies did not specify independent blind assessment of outcome.^5^, ^7^ Risk of bias for each outcome was assessed with the GRADE tool (Table S3). Appropriate and inappropriate shock had low risk of bias, as it involved objective ICD interrogation data with minimal reporting bias. Shock‐related physical injury had moderate risk of bias due to lack of randomization and possible undocumented minor injuries. Quitting sports due to shock had serious risk of bias, as the incidence was patient‐reported. Cardiac adverse events had moderate risk of bias due to selection bias and lack of randomization.

### 3.3 Study and baseline patient characteristics

Six studies conducted between 2013 and 2024 in the United States and Europe comprising 1,183 competitive athletes were included for the meta‐analysis.^6^, ^7^, ^8^, ^9^, ^10^ The studies comprised both retrospective and prospective cohort designs, with follow‐up durations ranging from 12.6 to 85 months. Patients were predominantly male (>50%). While not all studies reported the average age of participants, age criteria were clearly defined in the eligibility criteria of each study. Diagnoses among participants included a range of cardiac conditions, including long QT syndrome (LQTS), hypertrophic cardiomyopathy (HCM), catecholaminergic polymorphic ventricular tachycardia (CPVT), idiopathic ventricular fibrillation (IVF), arrhythmogenic right ventricular cardiomyopathy (ARVC), arrhythmogenic bileaflet mitral valve prolapse syndrome (ABiMVPS), Brugada syndrome (BrS), catecholamine sensitive ventricular tachycardia (CSVT), as well as structural cardiac conditions prone to arrhythmias such as dilated cardiomyopathy (DCM), valvular heart disease (VHD), coronary artery disease (CAD), congenital heart disease (CHD), and left ventricular non‐compaction (LVnc). One study by Johnson et al. exclusively included patients with genetically confirmed LQTS.^10^ Five studies were conducted on transvenous ICD, while one by Gasperetti et al.^6^ focused on novel S‐ICD. Further details regarding study and baseline characteristics are provided in Table 1.

**TABLE 1:** Study characteristics and baseline patient characteristics.

	Gasperetti et al.^6^	Tobert et al.^8^	Heidbuchel et al.^7^	Saarel et al.^9^	Lampert et al.^4^	Johnson et al.^10^
Study characteristics						
Year	2024	2022	2019	2018	2017	2013
Region	Europe, North America	US	Europe, US	US	US	US
Study design	Prospective cohort	Retrospective cohort	Prospective cohort	Prospective cohort	Prospective cohort (Research letter)	Retrospective cohort
Mean/median follow‐up duration, months	29.9 months (IQR 12.6–46.3)	46–85 months	43.9 (IQR 30.1–48.8)	42 months (IQR 25–40)	44 (IQR, 30–48)	61.2 month (IQR 32.8–77.9)
Participants, *n*	152	125	317	129	440	20
Loss to follow up, *n* (%)	3	20	0	27	37	0
Patient characteristics	Competitive, professional and leisure‐time athletes engaging in dynamic sports with subcutaneous‐ICD (S‐ICD)	GHD‐positive athletes indicated and implanted with transvenous ICD	Athletes with transvenous ICD aged 10–60 years	Patient aged 10–21 with GHD with transvenous ICD	Athletes with transvenous ICD aged 10–60 years	Patient aged 6–40 with genetically confirmed LQTS with transvenous ICD
Definition of athlete	Competitive athlete: one who participates in an organized team or individual soprts that requires regular competition against others as a central component, places a high premium on excellence and achievement and requires some form of systematic (and usually intense) training Professional athlete: competitive athlete for whom the practice of sports constitutes the main occupation and the principal source of incume Leisure‐time athlete: physically active person engaging in a range of exercise levels from modest to vigorous on a regular basis, not desiring to excel against others, and therefore not facing the same psychological and physical pressures as a competitive athlete	Individual who participate in competitive sports. Sports were classified according to Bethesda system of classification	Individual who participate in competitive sports with static and dynamic components greater than ‘IA’ defined by Bethesda system of classification	Individual participating in organized sports involving regular practice and regularly scheduled competition in sports that are typically classified as greater than category IA by Bethesda system of classification	Individuals participating in organized sports, involving regular practice and regularly scheduled competition with static and dynamic components greater than those traditionally classified as IA	Athletes participating in organised competitive sports at the professional, college, highschool, middle‐school or youth level, classified with Bethesda system of classification
Baseline characteristics of patients						
Male, *n* (%)	120 (79.0)	68 (54.4%)	216 (68%)	78 (60.5)	N/A	9 (45)
Age, years	38.4 ± 14.8	N/A	N/A	16.38 ± 2.73	N/A	14.5 ± 9
Time since initial ICD implantation, months	N/A	N/A	30.6 (12.0, 66.2)	18 (IQR, 9–40)	26 (IQR, 11–59)	N/A
Age at ICD placement, years	N/A	19.1 ± 10.6	37.7 (12.6)	N/A	N/A	N/A
Age at RTP, years	N/A	19.8 ± 11.6	N/A	N/A	N/A	N/A
Diagnosis, *n* (%)						
LQTS	4 (2.6)	56 (44.8)	47 (14.8)	49 (38)	87 (20)	20 (100)
HCM	14 (9.2)	22 (17.6)	48 (15.1)	30 (23)	75 (17)	N/A
CPVT	N/A	17 (13.6)	8 (2.5)	7 (5)	N/A	N/A
IVF	15 (9.9)	15 (12.0)	41 (12.9)	10 (7.8)	N/A	N/A
ARVC	17 (11.2)	8 (6.4)	43 (13.6)	6 (5)	55 (13)	N/A
ABiMVPS	N/A	3 (2.4)	N/A	N/A	N/A	N/A
BrS	29 (19.1)	2 (1.6)	7 (2.2)	1 (1)	N/A	N/A
CSVT	N/A	1 (0.8)	N/A	N/A	N/A	N/A
DCM	15 (9.9)	1 (0.8)	30 (9.5)	3 (2)	N/A	N/A
VHD	N/A	N/A	7 (2.2)	N/A	N/A	N/A
CAD	23 (15.1)	N/A	41 (12.9)	0	N/A	N/A
CHD	N/A	N/A	22 (6.9)	15 (12)	N/A	N/A
LVnC	N/A	N/A	5 (1.6)	2 (1)	N/A	N/A
None (family history)	N/A	N/A	5 (1.6)	N/A	N/A	N/A
Other	N/A	N/A	14 (4.4)	6 (4.7)	N/A	N/A
ICD indication	N/A	N/A			N/A	N/A
VF/cardiac arrest			103 (32.5)	39 (30.2)		
Sustained VT			54 (17.0)	8 (6.2)		
Syncope			75 (23.7)	40 (31)		
Prophylactic			74 (23.4)	36 (27.9)		
Positive EP study			11 (3.5)	18 (9–40)		
Family history of GHD	N/A	77 (61.6)	N/A	N/A	N/A	N/A
Family history of SCD	N/A	52 (41.6)	N/A	N/A	N/A	N/A
Total RTP‐ICD years of follow‐up, y	N/A	447.3	N/A	N/A	N/A	N/A
Average RTP‐ICD years of follow‐up, y	N/A	3.6 + −3.5	N/A	N/A	N/A	N/A
ICD rate cut‐off, beats/min	N/A		200 (188, 214)	214 (IQR, 205–222)	N/A	N/A
Monitoring		200–220				
Therapy zone		220–240				
VF zone		≥240				
Taking beta blocking agent, *n* ()	N/A	N/A	199 (65.3)	94 (72.9)	N/A	18 (90)
Hours of sports/week	N/A	N/A	6.0 (4.0, 10.0)	N/A	N/A	N/A
Shocks						
Appropriate	25 (16.5)	23 (18.4)	36 (11.36)	18 (13.95)	46 (10.45)	1 (5)
Inappropriate	19 (12.5)	6 (4.8)	29 (9.15)	5 (3.87)	2 (0.45)	0 (0)
Quitting sports due to shock	N/A	0 (0)	0 (0)	0 (0)	20 (4.5)	0 (0)
Shock‐related physical injury	N/A	0 (0)	0 (0)	0 (0)	0 (0)	0 (0)
Cardiac adverse events during or 2 h after sport	N/A	N/A	0 (0)	0 (0)	0 (0)	N/A

Abbreviations: ABiMVPS, arrhythmogenic bileaflet mitral valve prolapse syndrome; ARVC, arrhythmogenic right ventricular cardiomyopathy; BCE, breakthrough cardiac event; BrS, Brugada syndrome; CAD, coronary artery disease; CHD, congenital heart disease; CPVT, catecholaminergic polymorphic ventricular tachycardia; CSVT, Cathecolamine‐sensitive ventricular tachycardia; DCM, dilated cardiomyopathy; EP, electrophysiological; GHD, genetic heart disease; HCM, hypertrophic cardiomyopathy; ICD, implantable ardioverter defibrillator; IVF, idiopathic ventricular fibrillation; LQTS, long QT syndrome; LVnC, Left ventricular non‐compaction; RTP, return to play; SCD, sudden cardiac death; VHD, valvular heart disease; VT, ventricular tachycardia.

### 3.4 Meta‐analysis of outcomes

#### 3.4.1 Appropriate shock

**FIGURE 2:**
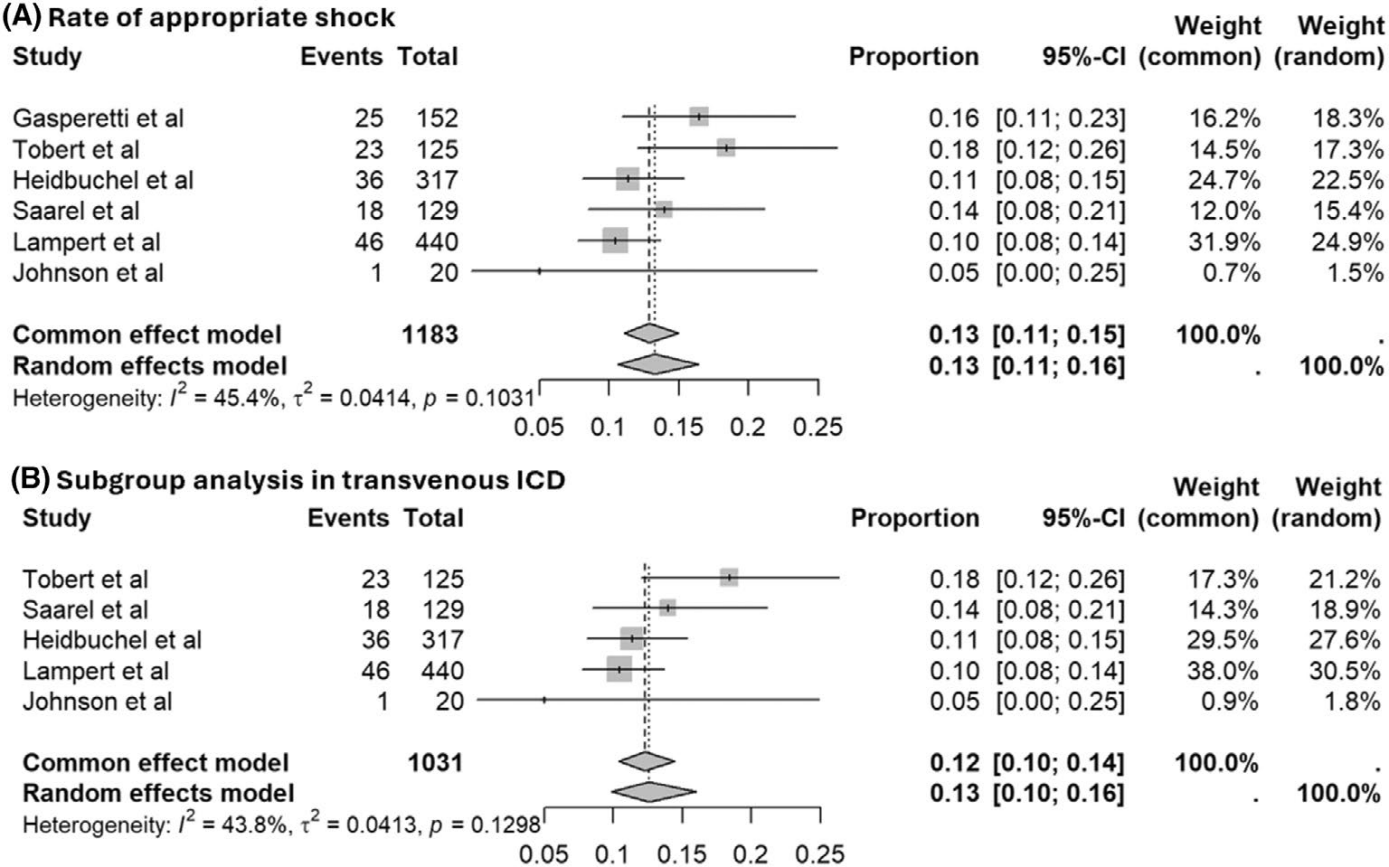
Forest plots of appropriate shocks in (A) all studies and (B) transvenous ICD only.

In the pooled analysis, we found that the rate of appropriate shock was 13% (95% CI: 0.11–0.16) with a low‐to‐moderate heterogeneity (*I* ^2^ = 45.4%) (Figure 2A). In the transvenous ICD subgroup, the rate was similar at 13% (95% CI: 0.10–0.16; *I* ^2^ = 43.8%) (Figure 2B).

#### 3.4.2 Inappropriate shock

**FIGURE 3:**
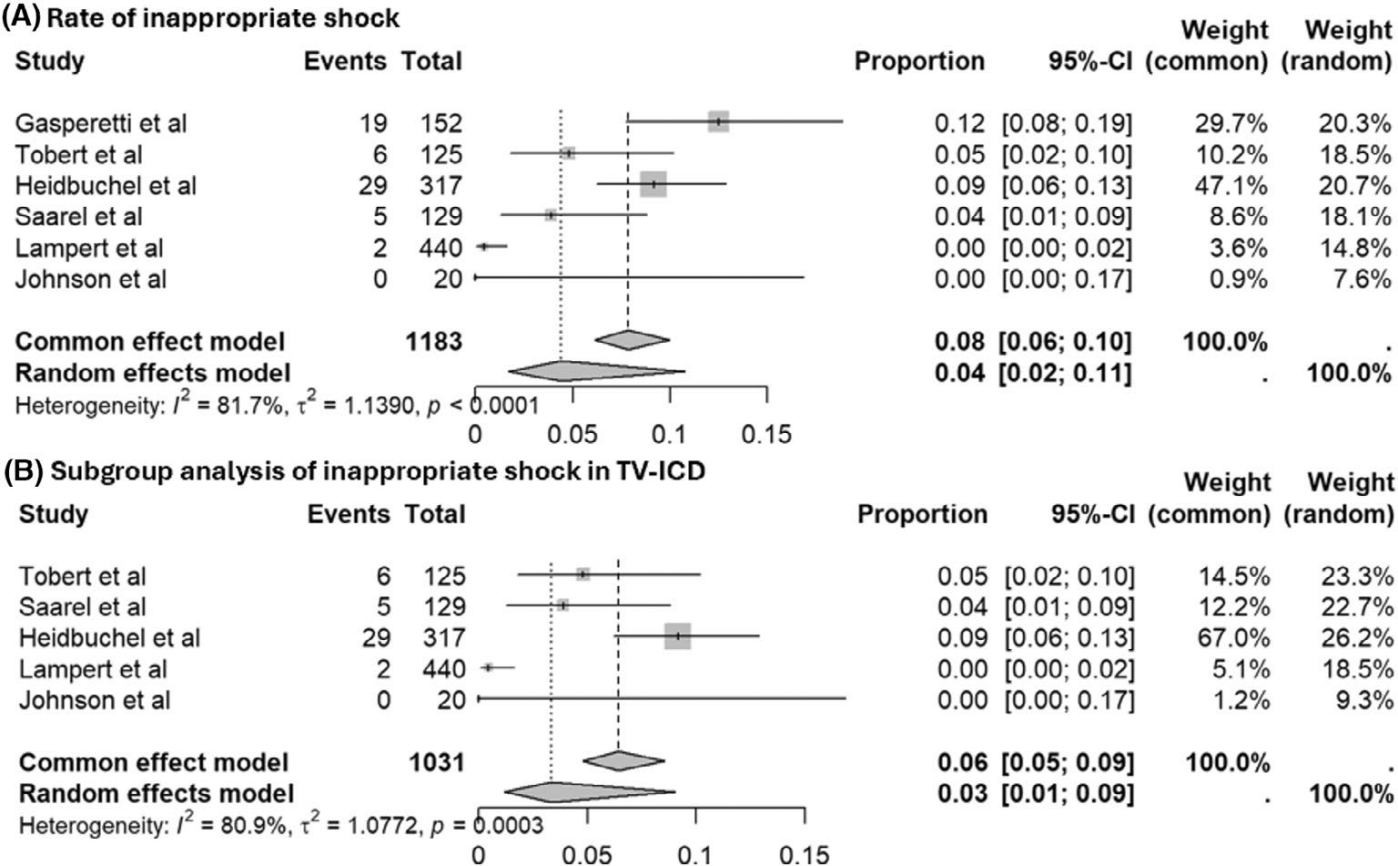
Forest plots of inappropriate shocks in (A) all studies and (B) transvenous ICD (TV‐ICD) only.

**FIGURE 4:**
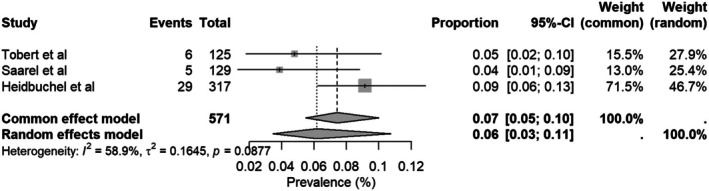
Forest plot of sensitivity analysis of inappropriate shocks.

The overall rate of inappropriate shock was 4% (95% CI: 0.02–0.11), with observed substantial heterogeneity (*I* ^2^ = 81.9%, Figure 3A). A subgroup analysis conducted in the transvenous ICD group showed a similar inappropriate shock rate of 3% (95% CI: 0.01–0.09, *I* ^2^ = 80.9%) (Figure 3B). For the sensitivity analysis, we excluded three studies with the lowest observed effect sizes^8^, ^9^ and the only study that evaluated S‐ICD.^6^ This resulted in an increased overall inappropriate shock rate of 6% (95% CI: 0.03–0.11), with a notable reduction in heterogeneity (*I* ^2^ = 58.9%, Figure 4).

#### 3.4.3 Shock‐related physical injury

**FIGURE 5:**
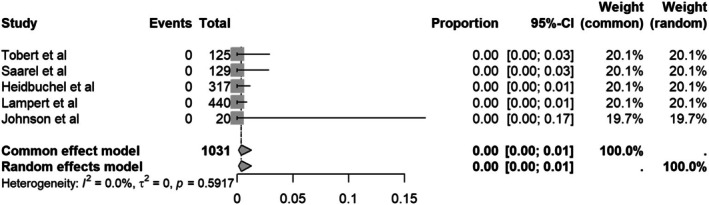
Forest plot of shock‐related physical injury.

From all the included studies, none (0%) reported events of shock‐related physical injury (95% CI = 0.00–0.01) with no heterogeneity (*I* ^2^ = 0%) (Figure 5).

#### 3.4.4 Cardiac adverse events

**FIGURE 6:**
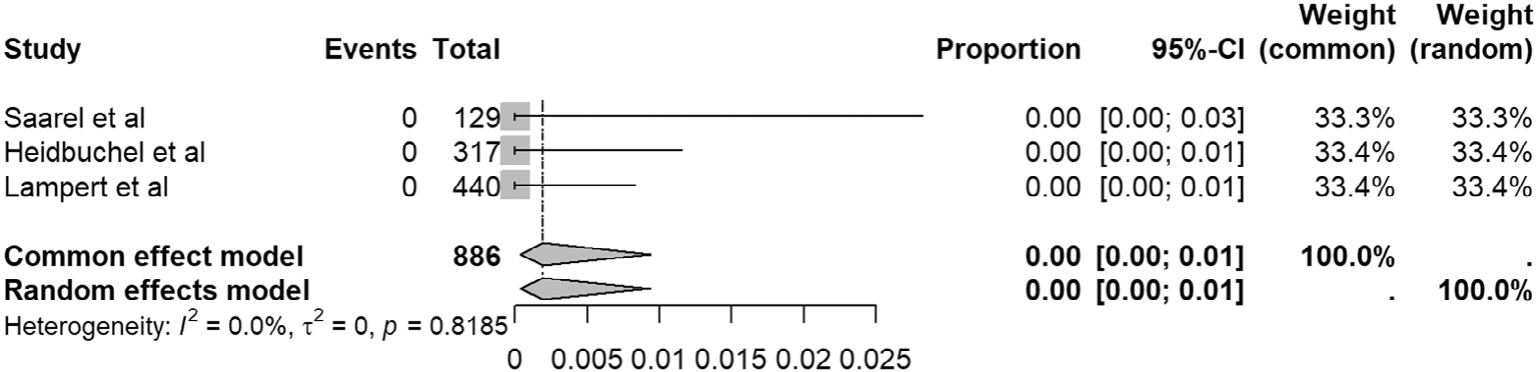
Forest plot of cardiac adverse events.

The rate of cardiac adverse event during or 2 h after sport is 0% (95%CI 0.00–0.04) with no observed heterogeneity (*I* ^2^ = 0%) (Figure 6).

#### 3.4.5 Quitting sports due to shock

**FIGURE 7:**
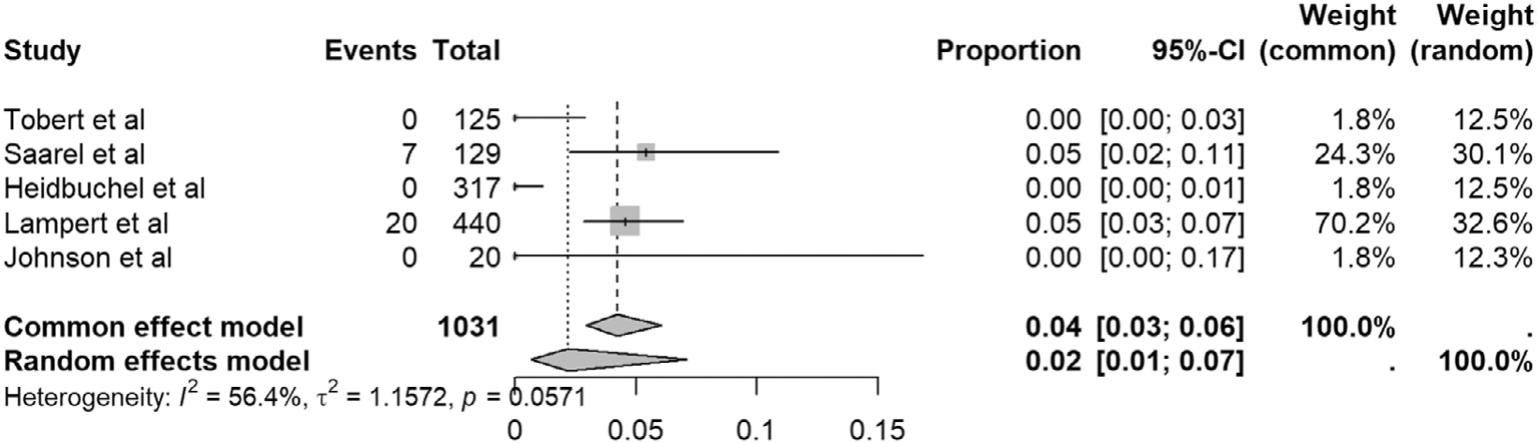
Forest plot of quitting sports due to shock.

**FIGURE 8:**
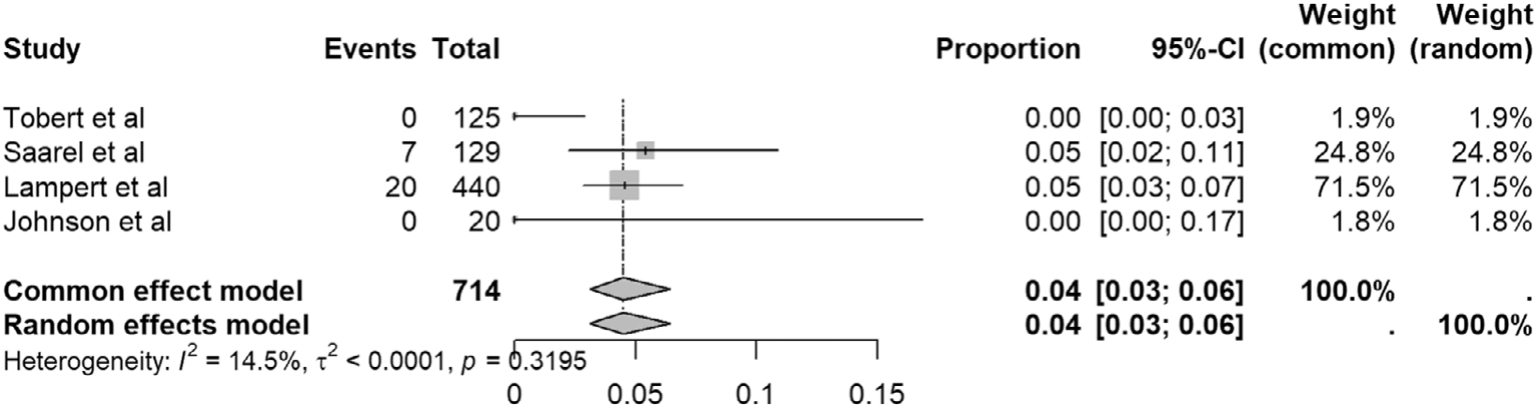
Forest plot of sensitivity analysis of quitting sports due to shocks.

The initial quitting sports due to shocks‐rate was 2% (Figure 7). Due to substantial heterogeneity (*I* ^2^ = 56.4%), we performed a sensitivity analysis using the leave‐one‐out method. Excluding the study by Heidbuchel et al.,^7^ which had the lowest 95% CI and a relatively large sample size, reduced heterogeneity to *I* ^2^ = 14.5%. This exclusion led to a slight increase in the pooled estimate from 2% to 4% (Figure 8).

## 4 DISCUSSION

This meta‐analysis provides an updated evaluation of the efficacy and safety of return‐to‐play in athletes with an ICD for congenital or acquired arrhythmias, including the novel S‐ICD.

The rate of appropriate shock in competitive athletes was 13% (95%CI = 0.11–0.16, *I* ^2^ = 45.4%), with no difference between the transvenous ICD and S‐ICD groups. Several studies have demonstrated that physical activity could trigger arrhythmias in individuals with pre‐existing cardiovascular conditions, and this could contribute to the shocks rate in athlete.^11^ While one retrospective cohort finding suggested that, compared to the general population, the rate of all shocks (appropriate and inappropriate) in athletes (defined as patients who exercised ≥5 h per week on a structural basis) was not more frequent compared to non‐athletes (25% vs. 33%, *p* = 0.760) and its appropriateness was not associated with the intensity of exercise,^12^ Heidbuchel et al.,^7^ studying European (EU) and US athletes, found that the rate of all shocks was significantly higher in competitive athletes compared to recreational athletes at any time and during physical activity or competition. The higher rate of appropriate shock during physical activity was bivariately related to underlying disease (mainly ARVC), secondary prevention indication, and competitive sports and was not associated with age, gender, beta‐blocker use, or burst vs. endurance sports. Although the total rate of shocks was higher in competitive athlete groups, the rate of appropriate shocks did not differ significantly (15.5% vs. 12.5%, *p* = 0.21), as the difference was found in the rate of inappropriate shocks (12.0% vs. 2.5%, *p* = 0.01).^7^ One more finding of this study is that competitive athletes received more shocks during activity while recreational athletes received more during rest, consistent with the paradox of exercise, where it could both trigger and protect against sudden death.^7^, ^13^ In a more specific population, LQTS athletes, Johnson et al. reported that the discrepancy of appropriate shock between athletes and non‐athletes was not observed, as there were no additional LQT‐triggered cardiac events among the athletes, on or off the athletic field, compared to LQTS patients who were not participating in competitive sports.^10^ These findings provide insight into the role of underlying arrhythmia as an important factor influencing the fate of athletes after ICD implantation, as the association between competitive sports and shocks rate remains in debate, particularly inappropriate shocks.

The rate of inappropriate shock was 4% (95% CI = 0.02–0.11, *I* ^2^ = 81.7%). After sensitivity analysis excluding three studies^5^, ^6^, ^10^ that contributed the most to the heterogeneity, the rate increased to 6% (95% CI = 0.03–0.11, *I* ^2^ = 58.9%). None of these events led to shock‐related physical injury (0%, 95%CI = 0.00–0.01, *I* ^2^ = 0%). The rate of inappropriate shock was reported to be higher in competitive athletes compared to recreational athletes (12.0% vs. 2.5%, *p* = 0.01), but this finding was not associated with increased sports discontinuation (3.8% vs. 7.5%, *p* = 0.32), a paradoxical finding. When compared to the general population, the rate of inappropriate shock appeared to be similar. In a retrospective nationwide cohort study utilizing data from the Danish ICD Registry (*n* = 4587), Ruwald et al. reported rates of appropriate and inappropriate ICD shocks of 17.6% and 4.7% at 4 years, respectively.^14^ Their study exclusively included patients with secondary prevention ICDs, which may explain the slightly higher shock rates compared to the findings of this meta‐analysis. Similarly, in a prospective, multicenter, nationwide study by Briongos‐Figuero et al. (the UMBRELLA study), the rate of inappropriate shocks at ±40 months was 6%.^15^ This study included patients with both primary and secondary prevention ICDs. Multivariate analysis identified a history of atrial fibrillation (AF) as a significant factor associated with an increased incidence of inappropriate shocks.^15^ One interesting and important finding from this meta‐analysis is that the rate of inappropriate shocks between the transvenous ICD and S‐ICD groups differs, as transvenous ICD was associated with a lower inappropriate shocks rate (6% vs. 12.5%), but the S‐ICD inappropriate shocks rate was acquired only from one study, highlighting the need for more study in this group in order to gain a more accurate and unbiased number. The number of inappropriate shocks in the S‐ICD group in athletes was similar to the previous study of S‐ICD in the non‐athlete population (12.5% vs. 13.1%).^16^

Rate of inappropriate shock is also affected by device programming and lead function.^17^ Garnreiter et al. reported that younger patients with slower ICD ventricular detection rates were more likely to receive inappropriate shocks. Additionally, several studies indicated that younger patients may possess a higher likelihood of experiencing inappropriate shocks or other device‐related complications. These issues are associated with factors such as size mismatch, somatic growth, frequent sinus tachycardia or supraventricular tachycardia (SVT), and the use of non‐standard hybrid or epicardial approaches for device placement due to technical challenges.^18^ This also applies to the adult population. When considering the difference between competitive and recreational athletes, the higher rate of inappropriate shocks among competitive athletes may be attributed to their greater susceptibility to sinus tachycardia. However, this can often be managed through device reprogramming, which may explain why it did not result in a higher rate of sports discontinuation. Thus, the Class I recommendation to program the tachycardia rate detection zone to 185–200 bpm to reduce total therapies may not be applicable to athletes. Athletes may benefit from a high‐rate cutoff (>200 bpm) and long‐detection duration using a personalized programming strategy, as recommended in the HRS 2024 consensus.^4^, ^18^, ^19^

Another important consideration is the preservation of lead function. Current studies suggest that lead function remained relatively well‐preserved even as athletes participated in high‐impact and endurance sports. The rate of freedom from definite lead malfunction ranged from 92.3% to 100% among the included cohorts.^5^, ^7^, ^9^ One study by Tobert et al., which included athletes with mostly (40.2%) class IIC sports activity (moderate static, high dynamic), reported that the overall rate of ICD device‐related complication, including device damage, inappropriate shocks, manufacturer recall, and secondary complications, was 29.6%.^8^ Most complications were secondary to device implantation (13.6%).^8^ All the complications were not directly related to any athletic activity.

The key question is whether the reported shocks were associated with serious adverse events. In this meta‐analysis, we found that the rate of cardiac adverse events during or 2 h after sport was 0% (95%CI 0.00–0.01, *I* ^2^ = 0%). However, the risk of cardiac adverse events during the return to play period is not negligible. Tobert et al., in a median follow‐up period of 3.6 + − 3.5 years, reported a 4% and 1.6% rate of syncopal events without shock but with recorded non‐sustained VT and ICD storm, respectively, recorded throughout their RTP period, but none occurred during sports‐related activities.^8^ Compared to non‐athlete GHD patients, one study by Rohatgi et al. reported that in LQTS patients, at the median follow‐up period of 6.7 years, the rates of arrhythmogenic syncope and ICD storm were 16% and 10%, respectively.^20^ The shorter follow‐up period in athlete GHD patients may contribute to the lower observed event rates compared to non‐athlete GHD populations. While the findings of this meta‐analysis suggest zero cardiac adverse events related to sport activities, further research with larger cohorts and extended follow‐up is essential to establish a more definitive conclusion.

Beyond the physical safety of returning to play with ICD, the psychological impact of ICD shocks during sports, whether appropriate or inappropriate, is essential to consider as a main contributing factor in sports discontinuation. Patients with ICD often experience emotional distress following ICD shocks, which has been associated with the likelihood of anxiety and depressive symptoms. In this study, we found that the rate of quitting sports due to shock was 2% (95%CI 0.03–0.06; *I* ^2^ = 56.4%). After sensitivity analysis, excluding one study with the narrowest confidence interval by Heidbuchel et al.^7^, which reported a 0% rate, resulted in reduced heterogeneity (*I* ^2^ = 14.5%) and increased the effect estimate to 4% (95%CI 0.03–0.06).^21^ Although the overall sports discontinuation rate in this study was 2%, among those who received shocks, several studies reported a 10%^5^ to 30%–40%^7^ rate of temporary sport discontinuation. While inappropriate shocks can often be addressed through prompt device reprogramming, the psychological impact of receiving a shock should not be underestimated. Therefore, ICD programming must be precise from the beginning, tailored specifically to the needs of athletes to minimize the risk of inappropriate shocks and prevent associated emotional distress. Furthermore, physicians should provide comprehensive support by not only optimizing device settings but also offering psychological counseling to help athletes cope with the potential risks and emotional burden of ICD implantation.

Considering all findings, ICDs appear to be effective and safe in preventing cardiac events, including electrical storm, syncope, hospitalization, and mortality, in athletes returning to play. However, the potential for increased appropriate or inappropriate shocks during competition remains a key factor in return‐to‐play decisions, highlighting the importance of shared decision‐making. These findings align with the latest 2024 HRS Consensus on Arrhythmias in Athletes, which grants a Class IIa recommendation for return‐to‐play in athletes with ICDs.^4^ The consensus emphasizes that athletes can safely resume competitive sports but also highlights the need for individualized risk assessment. While previous guidelines discouraged participation due to concerns over arrhythmia induction, device malfunction, and inappropriate shocks that may lead to hospitalization and mortality, recent evidence indicates that these risks may not be as restrictive as once believed, though careful individualized assessment remains essential. Current data suggest that transvenous ICDs might be safer than S‐ICDs, with a lower inappropriate shocks rate, but we believe more study on S‐ICD is needed to understand its safety better. Our findings further support assessing each athlete's age, underlying arrhythmia, comorbidities, and sports category, personalized ICD programming, and personal concern prior to the return‐to‐play decision instead of applying total restriction with shared decision‐making and clear discussion of potential risks.

## 5 STRENGTH AND LIMITATION

To our knowledge, this meta‐analysis provides the most up‐to‐date evaluation of implantable cardioverter‐defibrillator (ICD) use in athletes following the release of the 2024 Heart Rhythm Society (HRS) Consensus.^4^ However, several limitations should be acknowledged. Due to restricted access, we were unable to include EMBASE in our search strategy. To address this, we applied comprehensive search terms and broader keywords to enhance sensitivity. Nonetheless, the absence of EMBASE may have led to the omission of relevant studies.

The studies included in our analysis also possessed notable differences in baseline characteristics, particularly in the proportion of underlying arrhythmias and age distribution. This variability likely contributes to the substantial heterogeneity observed in several outcome measures. Additionally, all included studies were unrandomized, which is understandable since randomly assigning athletes and not giving them ICDs while they return to play could be harmful. The self‐selection in return‐to‐play decisions also adds to the study's limitations. However, despite these limitations, we believe this study provides important insight into the efficacy and safety of ICDs in athletes and was done as well as possible. In this context, the lack of randomization was unavoidable. While our meta‐analysis aims to provide a general perspective on ICD use in athletes, these findings should be interpreted with caution when applied to specific subgroups.

## 6 CONCLUSION

This meta‐analysis suggests that ICD use is relatively safe in athletes choosing to return to play, with no shock‐related physical injury and a minimal rate of serious adverse events. Increased rates of appropriate or inappropriate shocks, however, should be a key factor in deciding return‐to‐play. At the moment, transvenous ICD is associated with a lower rate of inappropriate shocks compared to S‐ICD. Optimizing ICD programming, including individualized detection thresholds and longer detection times, may further reduce inappropriate shocks and enhance safety. Rather than applying broad restrictions, our findings support a tailored approach, considering each athlete's underlying arrhythmia, comorbidities, the type of sport, training intensity, and personal concerns before making a return‐to‐play decision. Additionally, psychological support should be integrated into athlete care, as the emotional impact of ICD shocks plays a crucial role in sports discontinuation. More studies on S‐ICD in athletes returning to play are needed to understand its safety and efficacy better.

## CONFLICT OF INTEREST STATEMENT

The authors declare no conflict of interests for this article.

## Supporting information


**Table S1.** Search strategy.
**Table S2.** Risk of bias of included studies.
**Table S3.** GRADE Assessment of outcome.

## Data Availability

(1) Heidbuchel et al., 2019: https://pubmed.ncbi.nlm.nih.gov/30813818/. (2) Lampert et al., 2013: https://www.ahajournals.org/doi/10.1161/circulationaha.117.027828. (3) Johnson et al., 2013: https://pubmed.ncbi.nlm.nih.gov/23193325/. (4) Tobart et al., 2022: https://pubmed.ncbi.nlm.nih.gov/35985858/. (5) Saarel et al., 2020: https://www.ahajournals.org/doi/10.1161/CIRCEP.118.006305. (6) Gasperetti et al., 2024: https://www.sciencedirect.com/science/article/abs/pii/S1547527124033678.
