# Supplementary material for: Return‐to‐play in athletes with transvenous and subcutaneous implantable cardiac defibrillator: A meta‐analysis
Source: J Arrhythm. 2025 Jul 2;41(4):e70131. doi: 10.1002/joa3.70131 (PMC12222181; doi:10.1002/joa3.70131)
Supplement: Supplementary file 1 — Table S1. Search strategy. Table S2. Risk of bias of included studies. Table S3. GRADE Assessment of outcome. [file JOA3-41-e70131-s001.docx]

**Supplementary Table 1. Search strategy**

| Database | Keyword |
| --- | --- |
| Scopus | ( ALL ( athlete ) AND ALL ( implantable AND cardioverter AND defibrillator ) ) AND ( LIMIT-TO ( DOCTYPE , "ar" ) ) |
| Pubmed | "athletes"[MeSH Terms] OR "athlet*"[All Fields] AND ("defibrillators, implantable"[MeSH Terms] OR ("defibrillators"[All Fields] AND "implantable"[All Fields]) OR "implantable defibrillators"[All Fields] OR ("implantable"[All Fields] AND "cardioverter"[All Fields] AND "defibrillator"[All Fields]) OR "implantable cardioverter defibrillator"[All Fields]) |
| PMC | "athletes"[MeSH Terms] OR "athlet*"[All Fields] AND ("defibrillators, implantable"[MeSH Terms] OR ("defibrillators"[All Fields] AND "implantable"[All Fields]) OR "implantable defibrillators"[All Fields] OR ("implantable"[All Fields] AND "cardioverter"[All Fields] AND "defibrillator"[All Fields]) OR "implantable cardioverter defibrillator"[All Fields]) |
| CochraneLibrary | "athlete" AND "implantable cardioverter defibrillator" |

**Supplementary Table 2. Risk of bias of included studies**

| Articles | Selection | | | | Comparability | Outcome | | | Score |
| --- | --- | --- | --- | --- | --- | --- | --- | --- | --- |
|  | Representativeness | Selection | Ascertainment of exposure | Outcome at start |  | Assessment of outcome | Adequate follow-up length | Adequate follow-up |  |
| Gasperetti et al | + | + | + | + | + | + | + | + | 8 |
| Heidbuchel et al | + | + | + | + | + | - | + | + | 7 |
| Lampert et al | + | + | + | + | + | - | + | + | 7 |
| Johnson et al | + | + | + | + | + | + | + | + | 8 |
| Tobert et al | + | + | + | + | + | + | + | + | 8 |
| Saarel et al | + | + | + | + | + | + | + | + | 8 |

**Supplementary Table 3. GRADE Assessment of outcome**

| Outcomes | No. of studies | Study design | Risk of bias | Inconsistency | Indirectness | Imprecision | Publication bias | Quality |
| --- | --- | --- | --- | --- | --- | --- | --- | --- |
| Appropriate shock | 6 (Gasperetti, Tobert, Heidbuchel, Saarel, Johnson, Lampert)^5-9^ | Prospective and retrospective cohorts | Moderate (selection bias, lack of randomization, self-selection) | No serious inconsistency | No serious indirectness | No serious imprecision | No suspected bias | Moderate |
| Inappropriate shock | 4 (Gasperetti, Tobert, Saarel, Heidbuchel)^5-7^ | Prospective and retrospective cohorts | Low (objective ICD interrogation data, minimal reporting bias) | No serious inconsistency | No serious indirectness | No serious imprecision | No suspected bias | High |
| Shock-related physical injury | 6 (Gasperetti, Tobert, Heidbuchel, Saarel, Johnson, Lampert)^5-9^ | Prospective and retrospective cohorts | Moderate (lack of randomization, possible undocumented minor injuries) | No serious inconsistency | No serious indirectness | No serious imprecision | No suspected bias | Moderate |
| Quitting sports due to shock | 4 (Tobert, Saarel, Lampert, Johnson)^5, 7-9^ | Prospective and retrospective cohorts | Serious (patient-reported outcomes, possible unreported temporary sports discontinuation) | No serious inconsistency | No serious indirectness | Moderate (limited sample size) | No suspected bias | Low |
| Cardiac adverse events | 4 (Gasperetti, Saarel, Heidbuchel, Lampert)^-7, 9^ | Prospective and retrospective cohorts | Moderate (selection bias, lack of randomization) | No serious inconsistency | No serious indirectness | Moderate (limited sample size) | No suspected bias | Moderate |
